# Religio-cultural factors contributing to perinatal mortality and morbidity in mountain villages of Nepal: Implications for future healthcare provision

**DOI:** 10.1371/journal.pone.0194328

**Published:** 2018-03-15

**Authors:** Mohan Paudel, Sara Javanparast, Gouranga Dasvarma, Lareen Newman

**Affiliations:** 1 Southgate Institute of Health, Society and Equity, Flinders University, Adelaide, Australia; 2 College of Humanity, Arts and Social Sciences, Flinders University, Adelaide, Australia; 3 Education Arts and Social Sciences Divisional Office, University of South Australia, Adelaide, Australia; National Academy of Medical Sciences, NEPAL

## Abstract

**Objective and the context:**

This paper examines the beliefs and experiences of women and their families in remote mountain villages of Nepal about perinatal sickness and death and considers the implications of these beliefs for future healthcare provision.

**Methods:**

Two mountain villages were chosen for this qualitative study to provide diversity of context within a highly disadvantaged region. Individual in-depth interviews were conducted with 42 women of childbearing age and their family members, 15 health service providers, and 5 stakeholders. The data were analysed using a thematic analysis technique with a comprehensive coding process.

**Findings:**

Three key themes emerged from the study: (1) ‘Everyone has gone through it’: perinatal death as a natural occurrence; (2) *Dewata* (God) as a factor in health and sickness: a cause and means to overcome sickness in mother and baby; and (3) *Karma* (Past deeds), *Bhagya* (Fate) or *Lekhanta* (Destiny): ways of rationalising perinatal deaths.

**Conclusion:**

Religio-cultural interpretations underlie a fatalistic view among villagers in Nepal’s mountain communities about any possibility of preventing perinatal deaths. This perpetuates a silence around the issue, and results in severe under-reporting of ongoing high perinatal death rates and almost no reporting of stillbirths. The study identified a strong belief in religio-cultural determinants of perinatal death, which demonstrates that medical interventions alone are not sufficient to prevent these deaths and that broader social determinants which are highly significant in local life must be considered in policy making and programming.

## Introduction

Poor perinatal health is a global public health problem and reflects issues of inequality and injustice. Perinatal deaths include both stillbirth and neonatal death [1, 2]. Worldwide, over 14,500 perinatal deaths occur each day [3, 4] almost 99% of them in developing countries. Among developing countries, Sub-Saharan Africa and South Asian countries record over three-quarters of the world’s stillbirths [5] and neonatal deaths [6]. Furthermore these regions have experienced slow progress in reducing perinatal mortality rates in the past two decades [7]. With only a 2.7% annual rate of reduction in neonatal mortality rate (NMR) between 2000–2012, it is estimated that it will take 110 years for an African and about 80 years for a South Asian woman to experience a similar chance of saving their newborn baby as is currently experienced by the average woman in developed countries [8]. Moreover, at current rates of reduction of the stillbirth rate, about 160 years and 100 years will pass before the average pregnant woman from Africa and South Asia respectively experience the same stillbirth rates as a woman in a developed country [9]. These differences are principally attributed to the weak health systems and poor coverage of maternal and neonatal care provision in Africa and South Asia [10].

National statistics from Nepal show that, despite significant progress over time, the rates of neonatal and perinatal deaths are still high at 33 per 1,000 live births and 37 per 1,000 live births respectively [11]. Within Nepal, significant disparities exist in the distribution of perinatal mortality rates. The mountain region has a neonatal mortality rate of 46 per 1,000 live births, with 85% of all neonatal deaths occurring within the first week after birth [11], while the perinatal mortality rate is likely to be higher due to the underestimation of stillbirths. These figures are equivalent to the highest mortality rates in Sub-Saharan countries. Nepal’s national policies on maternal and child health acknowledge equity, rights-based approaches, family/community based care, woman-friendly, 24 hour birthing services, social inclusion in maternal and newborn care, and counting every perinatal death [12–16]. However, strategies developed to tackle poor perinatal health are predominantly medically-oriented and emphasise the treatment of newborn infections and promotion of health facility births. Studies undertaken in Nepal are also focussed on medical causes of perinatal deaths [17–20] and on epidemiological investigations of socio-demographic and health service utilisation variables and their distribution across the country [21, 22]. There is little evidence examining the social determinants of poor perinatal health, particularly in the country’s most disadvantaged areas.

The literature suggests that biological events such as pregnancy and childbirth are also socio-culturally constructed and therefore we need to understand tradition, society and culture and to examine the socio-cultural contexts of pregnancy and childbirth [23–26]. Traditions, social values and culture shape pregnancy and childbirth experiences and have a strong impact on women’s choice and control over both maternal and child health [23]. Values and cultures are of course different in different societies [25]. Perinatal deaths are linked to social, cultural and religious beliefs and values. For example, Hmong women living in Australia believe that disharmony in personal health as well as in the supernatural world causes miscarriage, stillbirth and neonatal death [27, 28]. Likewise, studies from Ethiopia, Tanzania, Uganda and Ghana found similar perceptions among women and their families about neonatal and infant death. In Ethiopia, supernatural forces are believed to cause perinatal death and dead babies are often buried in the house or in the backyard without any notification of birth or death [29]. This Ethiopian study identified that it is not permitted to mourn a perinatal death as it is considered to be against God’s will. Similar findings are reported in Uganda [30] and Ghana [31].

A range of studies discuss religious affiliations related to, or associated, with both positive and negative health outcomes [32–36]. The impact of religion on health is described mainly through two pathways: by affecting individual beliefs and norms related to health practices, and by increasing people’s social capital/connectedness and empowering people to effectively utilise relationships and resources for their health and wellbeing. Positive health outcomes are described mainly in supporting people to live through and cope with the tragedies of bereavement, HIV/AIDs, non-communicable diseases such as cancer, positive mental health and reduced addiction/substance abuse and crime. Negative outcomes are about not seeking, or delayed seeking of, medical care due to rigid religious beliefs.

Lay beliefs and lay knowledge have proven crucial to understanding and addressing the social determinants of health [37–41]. Lay beliefs offer an explanation for what people do and why they do it (including health behaviour), which could contribute to more effectively planning or tailoring health policy or interventions. Although the data highlight that perinatal mortality is high in Nepal’s mountainous region [11], no study to date has examined women’s and families’ views about poor perinatal survival within the broader sociocultural contexts. Studies conducted in Nepal mostly cover the plains or semi-urban hilly regions, and are often limited to describing patterns of mortality or service utilisation [42–48], or medical causes of perinatal deaths [49–53]. An in-depth socio-cultural understanding of what lies beneath the continuing high perinatal mortality rates is lacking both at the national level and more specifically for the mountainous region which exhibits the highest rate. This paper aims to explore the beliefs and experiences of local communities about perinatal sickness and death in these mountainous villages.

## Methodology

The study was conducted in two rural mountain villages of Nepal which rank the lowest on the Human Development Index (0.304), have one of the worst reported child survival rates in the country, and have no access to transportation. The two mountain villages provided a diversity of context: one village is predominantly *Khas* ethnic, called Aryans and who follow Hindu religious beliefs, with access to two village health facilities (a community birthing unit and a health facility), district hospital, and the other village comprising mostly *Lama* people of Tibetan descent who follow Buddhism, and have access to a village health facility with a birthing unit. This selection enabled the principal researcher to conduct fieldwork for data collection within the constraints of practicality in such remote areas. This study does not intend to compare the villages as such, rather the different villages were chosen to present findings as a case about how women and families living in remote mountainous settings explain and deal with perinatal deaths. Another reason for choosing the two villages was to reach the required number of participants in this study—the women and families who had recent perinatal deaths.

Qualitative interviews were conducted between February and June 2015 with 42 women and their families who had experienced a neonatal death or stillbirth in the previous four years, nine Skilled Birth Attendants (SBA), two Female Community Health Volunteers (FCHV), two support staff, one Auxiliary Health Worker, and five other stakeholders **(S1 File).** Women interviewed were in the age range 16 to 35 years. Women continued to be interviewed until a saturation point was reached where no new information was obtained by interviewing additional women. This saturation point was reached after interviewing these 42 women. Separate interview guides were developed for the women and families, the health service providers, and the other key stakeholders **(S2 File).** The stakeholders comprised local journalists and staff of non-governmental agencies working in the field of maternal and child health, and child marriages. The views and experience of health service providers of their day to day experiences about provision of health services to improve mother and baby’s survival, and that of the local stakeholders, supplemented the data from women’s interviews and helped to understand a comprehensive picture of ongoing perinatal deaths in the study communities. Interviews covered a range of questions around mothers’ experience of perinatal death and sickness. Participants were identified purposively using local volunteers, FCHVs and through contact with health facility staff.

Interviews were conducted in the local language by the first author and audio recorded with participants’ consent. The first author was assisted by the local health volunteers and a health service provider during interviews with participants from *Lama* Communities. Although *Lama* women and their families spoke Nepali, it was felt that helping them to speak in their own local language would encourage them to more naturally explore the phenomenon—contexts of perinatal deaths. The first author is a Nepali national with seven years’ experience as a health worker in Nepal’s hilly and mountain regions.

The study was approved by the Social and Behavioural Research Ethics Committee of Flinders University, the ethical board of Nepal Health Research Council, and the District Health Office of the study district in Nepal. Written informed consent was sought from the participants before the start of the interview.

### Data analysis

Interview files were simultaneously translated and transcribed into English by the first author. Six random transcripts were checked by five bilingual (Nepali and English) experts to ensure consistency in transcription and translation into English. NVivo version 10:00 software was used to organise the data and facilitate the development of coding frames. The texts were analysed with a comprehensive coding process, using an inductive thematic analysis technique as suggested by Braun & Clarke (2006). Thematic analysis involves identifying, analysing and reporting various themes from the data, where themes are the central organising concepts about the data. In this study, these are the aspects within the data which have revealed the socio-cultural contexts influencing perinatal deaths in the remote villages under study.

## Findings

The analysis of data provided three key themes emerging from the qualitative interviews in relation to religio-cultural contexts that describe predominantly the views of acceptance and fatalism about perinatal death and sickness. These themes are: (1) ‘*Everyone has gone through it’*: *perinatal death as a natural occurrence*; (2) *Dewata (God) as a factor in health and sickness*: *a cause and means to overcome sickness in mother and baby*; and (3) *Karma (Past deeds)*, *Bhagya (Fate) or Lekhanta (Destiny)*: *ways of rationalising perinatal deaths*.

### 1. ‘Everyone has gone through it’: Perinatal death as a natural occurrence

The collective experience of perinatal death among the study participants, their families, neighbours and the whole community has contributed to the notion of perinatal death as a natural and acceptable occurrence. This notion is expressed as follows by a 20-year old mother with experience of losing her child:

My grandmother had 10 births (Sutkas—childbirths). All of her babies died…., after 12 years she delivered my father and his three sisters. My father says, ' you are young, you can bear babies, you haven't lost anything'. My father-in-law is also the only surviving son in his family. (Interview, mothers)

Another participant, while describing her own and her sister’s story, labelled perinatal death as a common phenomenon that everyone faces:

My sister lost two babies, a boy, and a girl. We both lost our babies. Now she has two living children, and I have four. Everyone has gone through it. We can do nothing about it… (Interview, mothers)

Perinatal deaths, particularly for the first or second pregnancies, have been seen to occur in the participants’ generation, their parents’ generation, their grandparents’ generation and so on, and have been accepted as intergenerational life events. This has led to a perceived lack of control over mother and child health. A local auxiliary nurse also confirmed that the village women take the loss of a baby rather naturally, as a ‘generational continuum’:

Village women believe that losing a baby is seen as a generational perpetuation in their families. They say, 'my sister also lost one, my mother-in-law had the same experience and my mother had also lost babies. (Interview, service providers)

The commonness of the experience of perinatal loss is reinforced further when women and families find local Female Community Health Volunteers (FCHVs) and health service providers also experiencing similar events:

That female health volunteer (she points towards a nearby female health volunteer’s house) lost a son after birth. And, the two women over there also lost their babies. It is like this here, it occurs with everyone. (Interview, mothers)

#### 1.1 Timing and perinatal deaths: Low level of concern about early life

The study revealed that there is an association between the time of death and the level of acceptance: the sooner the baby dies, the more acceptable it is for parents and community. Stillbirth and death immediately after birth or during the mother’s confinement in *Gotha* (cowshed) is readily acceptable:

I didn't feel worried about the stillbirth (Hudaimareko). I felt it was okay. It rather made me easier to resume my day to day work sooner. (Interview, mothers)

*Gotha* is the place of birth, the ground floor of their house where a woman gives birth and resides during the postnatal period until about three weeks after birth. This study explored that both mother and baby are considered impure *(Chhuhi)* after birth. To keep the main part of the house pure, and not to pollute their *Dewata* (God), the birthing mother and her baby remain confined in *Gotha*. By adhering to this tradition it is believed that they are not displeasing their God, and hence protecting themselves from any harm that might result from God’s wrath.

It is not a matter of significant concern when a baby dies before the last month of pregnancy (*Hunemahina)*. In the local dialect, the term foetal death is used interchangeably with miscarriage which shows the lack of special importance given to pregnancy loss.

My wife had a foetal death (Aadanjhadne), but that was not a death, she lost it at six months of pregnancy. (Interview, mothers)

The concern towards a baby’s death increases when a woman enters the main home after *Gotha* around three weeks after birth, and family members visit the baby. In these mountain areas, perinatal deaths have no ritual significance. From the viewpoint of health volunteers, the repeated occurrence of perinatal deaths and lack of social or religious concern placed on them render them not worth counting or reporting. Foetal death was also described by participants as *Pakhala—*literally translated as a diarrhoeal disease—which metaphorically means a non-significant, common and natural occurrence. This perception about foetal death is shown in an interview with a Female Community Health Volunteer (FCHV):

There are many women losing their babies in pregnancy (Pakhalajane) and neonatal deaths. You can see these in every single house. Are we going to record all such deaths? Two years ago, even the local doctor’s (Auxiliary Health Worker) sister-in-law lost her baby. I know two women there who lost their babies in pregnancy (Pakhala). A woman in that house [she points to the house] had twin babies, but both died as newborns. (Interview, health volunteers)

Poor recording/reporting of such deaths became obvious when the first author on fieldwork reviewed the health facility records and had day to day conversations with the FCHVs during the recruitment of participants to this study. Moreover, it was not easy to get the data at first. Initially, FCHVs could not remember any such death in their neighbourhood. Over time, during repeated contacts and conversations, they started recalling women who had experienced perinatal losses. During the five months of fieldwork in the two study villages, eventually 42 women voluntarily reported 49 perinatal deaths (16 stillbirths, and 33 neonatal deaths) occurring in the last four years, with a majority of them occurring in the last two years. These 42 women are estimated to represent approximately 3% of the women of childbearing age in the study villages. By comparison, the local health facilities in the two study villages reported only five neonatal deaths in their verbal reports and only three in the local records. Not a single stillbirth was reported in the village health facilities. Based on these crude data, it is estimated that the study villages have a current neonatal mortality rate of 44 per 1,000 livebirths–similar to the official estimation of 46 per 1,000 as reported in the 2011 Nepal Demographic and Health Survey. This field visit also revealed an extended perinatal mortality rate of 63 per 1,000 births if stillbirths and all neonatal deaths until 28 days after birth are included in the calculation [54]. These estimates are based on the reported number of perinatal deaths for the previous four years. It should be noted that these two rates–neonatal mortality rate and the extended perinatal mortality rare–are not strictly comparable because the neonatal mortality rate is based on live births but the extended perinatal mortality rate is based on all births (live births plus still births). With a household survey based on a representative sample, the rates would be likely to be different because women in this study were participants of qualitative interviews who willingly reported their perinatal losses.

### 2. The will of *Dewata* (God) in health and sickness: A cause of, and a means to overcome sickness in mothers and babies

The word *‘Dewata’* is a collective name of Hindu Gods and Goddesses. The study revealed that believing in God’s will as the cause for health and sickness has a very strong impact on views around perinatal death. One can find the *Dewata* represented in various symbolic forms everywhere: for example, as statues, temples and ribbons in the farmland, on the banks of streams, on street corners, in the forest, inside the houses and in the middle of the villages.

#### 2.1 *Dewata* (God) and childbirth complications: Seeking faith healers during pregnancy, and childbirth

God’s will is believed to be a key cause of problems in pregnancy and childbirth. One of the participants, a 22 year old mother, commented that her breech presentation during delivery and the subsequent neonatal death were due to not worshipping their God:

My baby did not die due to breech presentation (Ulto). It is because God (Dewata) was angry with us [she cries]. My family should have called the faith healer (Lama), and prayed to God [Gyana]. They didn't do anything to please God at home [she cries], therefore my baby died. Local faith healer had told that it was not going to be a good fortune if we didn’t worship God. (Interview, mothers)

It is believed that faith healers can make the necessary prayers to please God. Faith healing is an old tradition practised for generations and has a strong foothold in these villages. Faith healers outnumber health volunteers and health service providers. There are different cadres of faith healers locally known as *Dhami-Jhakri*, *Dangri* in the *Khasan* community; and *Lama*, *Chumba* in the *Lama* community. Women and their families believe faith healers are chosen by God, they can understand God’s language and can alleviate any kind of suffering including women’s sickness. The faith healers are usually called to be present during childbirth and they are relied upon to relieve a woman from pain and suffering, to speed-up the birthing process and to save the lives of mothers and babies. They are also relied upon to help with other family health issues and pregnancy complications such as fainting during pregnancy; prolonged and severe labour pain; breech position of the baby; and when a woman feels weak during the birthing process.

**2**.**1**.**1 God’s will and impact in health care seeking**

Seeking assistance from professional health providers is often not the first choice of treatment. Professional health providers are sought only when the faith healers fail to provide assistance and if the faith healer gives the women and their families permission to contact a health provider. A local health worker described a moment when he had to attend a woman in labour at home together with a faith healer:

The faith healer (Lama) was ringing a bell around and reciting prayers (Mantras). I had to deliver her by rupturing her membrane. They invite us only when they are permitted to do so by the faith healers. (Interview, service providers)

Believing any sickness is a result of God’s will means that formal health care for any health problems during pregnancy and childbirth is rarely sought. A local Auxiliary Nurse confirmed this:

She [referring to a pregnant woman] was seven months pregnant. I asked her family to describe to me what had happened. They said that she died due to God's curse (Dewatalagne). (Interview, service providers)

#### 2.2 *Dewata* (God) after birth—seeking and receiving care for babies

Women and families also attribute their babies’ sickness to God’s displeasure. In their daily conversations, the range of sicknesses are together called ‘God’s wrath (*Dewatalagne)’*. It is believed that sicknesses happen when God is not pleased. To alleviate the sicknesses, one is expected to please God by prayers, wearing amulets, sacrifice (animal) and offerings made through faith healers.

**2**.**2**.**1 Local sickness labels**

The different sickness types frequently described by the participants are listed in Table 1.

**Table 1:** Sickness types reported by women and families in the villages.

**Sickness types**	**Beliefs**
**Type 1 Sickness: God as a cause and alleviator/cure of sickness**	
• Influence of the forest God (Lasolja/ Ban Dewata); • Influence of the God of the parent’s home *(Maiti Dewata)*, God of the in-law’s home *(Poili Dewata)*, and God of a family clan *(Kul Dewata)*; • Astrological hindrance *(Grahalagne)*; • Influence of a ghost *(Bhut/Lago/Masanlagne)*; • Witchcraft, evil eye *(Boksolagne/Koptini)*; • Influence of dead spirits of family or close relatives *(Muiyalagne)*; • Influence of the snake God *(Naglagne)*;	God is a major cause of sickness, and pleasing God is believed to be the key solution to alleviate sickness. Faith healers are sought for prayers, Mantra recitations, offerings (animal sacrifices and others), provision of amulets etc.
**Type 2 Sickness- God as a cause, with alleviation/cure through God and local traditional therapy**	
• Continued baby loss related to weakness of womb *(Mojhlagne)*; • Effects of heat with symptoms of diarrhoea and fever *(Taaplagne)*; • Cough and cold *(Sardilagne)*; • Pseudo teeth in baby’s neck *(Chordat)*; • Sudden rainbow attack and death *(Banlagne)*.	God is attributed as a cause, yet care is combined with local herbs. Faith healers who practise both faith healing and herbal practices are sought. These are characteristics of infections (diarrhoea, pneumonia, tetanus in babies) and anaemia and malnutrition in women.

**Type 1 sickness**: **God attributed as a major cause and a means to alleviate/cure sickness**

The type 1 sickness labels in Table 1 were attributed mainly to God’s will. Participants believed that to overcome these sicknesses they need to please their God. Faith healers were utilised to make offerings to God and to make their God happy. For example, a new mother’s lack of breastmilk was believed by one family to be caused by witchcraft/evil eye, for which they called a faith healer:

For three days, there was no milk secreted from her breasts. We contacted faith healers (Dhami, Dangri) to avoid witchcraft ‖; four faith healers (two Dhamis and two Dangris) came to our home. …We could not save him [the baby] longer; he died on 27^th^ day. (Interview, family members)

Likewise, for another participant, her newborn with complaints of vomiting was believed to be afflicted by a ghost *(Bhut)* and the forest God (*Bandewata)*. She lost her newborn baby last year:

We contacted local faith healers (Dhami and Lama). They said the baby was under the influence of a ghost (Bhut). They also told me to pray to the forest God (Bandewata). But, the baby died early. I couldn't even manage to pray to the forest God. (Interview, mothers)

In addition, participants believe that pregnant or postnatal mothers become unwell if they attract the wrath of God while on the way to, or working in the forest for grazing cattle and collecting fodder, firewood and grass. They believe that the forest God could affect a baby in the womb.

The God of a family clan *(Kul Dewata)* and astrological hindrance *(Graha*), which is the influence of heavenly bodies, is also believed to inflict sickness on mothers and babies. After her three perinatal losses (1 stillbirth and 2 neonatal deaths), one interviewee mother described how her family worked to make their God happy to save babies:

We worshipped to overcome astrological hindrance (Graha), contacted faith healers (Dhami) from around the villages to know why I continued losing my babies. We worshipped the God of my parents’ home (Maiti Dewata) as well as the God of my in-laws’ home (Poili Dewata). We tried our best to worship and pray to God. I don’t know why I continue to lose my babies. (Interview, mothers)

Women believe that the unhappy God of their family clan *(Kul Dewata)* could bring disgrace to the land and the site of their house. One mother also consulted the faith healer when her newborn baby was bleeding from the umbilicus. However, her baby died on the 11^th^ day after birth:

Both the faith healers (Dhami and Lama) were right about me; there is something wrong with this house [the site of the house]. I lost my two children here. They said, 'God (Dewata) of this house is unfavourable to you'. Like they said, both my children died at this house. (Interview, mothers)

They also believe that an angry God can affect people through the hungry dead spirits *(Muiya)* of the deceased family members or close relatives. A mother-in-law described the deaths of her two grandchildren (newborn babies) from this cause:

It was not a sign of luck. We contacted local faith healers (Dhami, Dangri) to offer the spirit (Muiya). Yet the baby did not survive. Nothing worked. (Interview, family members)

The health seeking behaviour of families is strongly influenced by their beliefs about God’s will in disease and death. For an unhappy God, the medicine from a health facility is perceived to have no effect, hence participants are reluctant to contact health workers and believe that seeking care from a health facility could even be harmful. A young husband aged 18 years stated:

Here in our tradition, if it [disease] is due to God, medicine doesn't work at all. If it was due to God, and they took medicine, it would further harm. (Interview, family members)

**Type 2 sicknesses**: **God as a cause, and sickness alleviated through God and local traditional therapy**

Women and families refer to God as a main cause of sickness, yet sometimes they seek combined care including herbs from local herbalists, worship, prayers and Mantra recitations from the faith healers (Table 1). They seek local herbalists, called *Baiji* to overcome sickness, particularly when the sickness types are perceived to be *Taplagne (effect of heat)*, *Banlagne (rainbow attack)* or *Mojhlagne (weakness of the womb)*. In these sickness types, God is attributed as a main cause, and they prefer to seek a faith healer and herbalist together. Even if it is the herbal medicine from *Baiji* (herbalist), they still focus on pleasing God by sacrificing animals and making offerings. A baby is perceived to suffer from *Taplagne* when found hot (feverish), and having diarrhoea or vomiting:

Taplagne makes their body hot and febrile, causes diarrhoea (Chherne), vomiting (Ukhalne), and Pneumonia (Sardi). A faith healer, who practices both as a faith healer and a herbalist (Baiji) treats baby with local herbs, throws holy grains (rice) and water over the sick baby’s body, and prays and worships God. Pastes made from herbs are applied on the baby's head and body. (Interview, mothers)

A sickness is attributed as *Banlagne (rainbow attack)* when they do not have any other explanation for babies’ deaths, such as death soon after birth. One of the participants described:

My neighbour also lost her baby boy due to Banlagne (rainbow attack). Experienced herbalists can treat it. We need a broom, Khukuri (a knife) and a bird feather (Garud) to worship God to alleviate Banlagne. (Interview, mothers)

When a woman is believed to be suffering from *Mojhlagne (weakness of the womb)*, she is considered more vulnerable to continuous baby losses in pregnancy or soon after birth. The participants believe that this occurs due to God’s curse, and believe that it can be treated by transferring it to specific plants or fruit trees with the help of an experienced faith healer and herbalist.

#### 2.3 Perinatal losses: Repeated pregnancies and aversions to family planning—God’s will?

Women suffering perinatal losses and stillbirths go through many pregnancies. In the remote mountainous region of Nepal, where the women are often poor, malnourished and less educated, such repeated pregnancies at short intervals are liable to end in stillbirths, or perinatal deaths if the pregnancy goes to term [55–58].

The use of contraceptives that can prevent frequent pregnancies and consequently may assist in preventing perinatal deaths is believed to be against God’s will. This is particularly related to vasectomy, the male method of sterilisation. Participants believe that if they have a vasectomy, their God will be unhappy and bring bad luck to their family, such as disease or death of family members; this includes pregnancy loss and newborn death, as well as damage to livestock and property:

I am scared. No one has done it [vasectomy]. If you do it before you have chosen to invoke Dewata (God), that might be okay. After God comes into you [after invoking God and becoming a faith healer], it is not good. It can go wrong. …You never know. You might encounter any bad consequences of it. I am afraid if I become further sick [although I am not a faith healer]. (Interview, family members)

One mother, aged 32 lost seven children (including stillbirths, neonatal, infant and toddler deaths) out of her 10 pregnancies. Her husband shared his fear of vasectomy, believing that his family God (the *Kul Dewata)* does not favour it.

…because of our family God (Kul Dewata), the operation [vasectomy] won't suit me. My father had the operation, then this didn't work, he died when he was just 42, very young. He was very young. (Interview, family members)

It is to be noted that ongoing perinatal deaths in these areas are also associated with gendered expectations about a girl and a daughter-in-law. Young girls are viewed to be secure and settled in the in-laws’ family by giving birth to a baby who lives, preferably a baby boy. The context of high mortality in the study areas exerts a pressure to repeatedly conceive. Hoping to have a baby who lives, preferably a baby boy, young women have repeated pregnancies with shorter birth intervals. The weak social position of a daughter-in-law (pregnant woman) renders them powerless to decide on how many children to bear and when. They are often controlled by their mothers-in-law and husbands about pregnancy and childbirth matters.

### *3*. *Karma* (past deeds), *Bhagya* (fate) or *Lekhanta* (destiny): Ways of rationalising perinatal deaths

According to the Oxford dictionary, the Hindu or Buddhist notion of *Karma* is defined as “the sum of a person’s actions in this and previous states of existence, viewed as deciding their fate in future existences”. The Indian scholar, Krishan [59] described *Karma* in the Hindu religious sense as an ‘action potential’ manifesting into a result or consequence which influences an individual during her/his next life. Likewise, the nearest English translation of *Bhagya* refers to fate. Similarly, the English translation of *Lekhanta* means ‘already written’ or ‘pre-destined’.

In these study villages, *Karma* or fate as reasons for perinatal death is attached personally to a woman’s *Karma* or fate. It is believed that one’s good Karma in the past yields good results. If one had bad *Karma* in the past, the results would be bad, which s/he has to experience during the present lifetime. One 35 year-old’s story, who is currently pregnant with her 10^th^ child, shows how she related Karma and fate to her repeated pregnancies and the deaths of her babies:

I lost these babies [her three children]. Had they survived, why should I have had too many births? I am unlucky, this is my fate (Bhagya); this is my Karma. (Interview, mothers)

By *Bhagya* (fate), the participants in this study referred to the current state that a woman has been facing, and which is perceived as a result of one’s *Karma*, therefore the word *Bhagya* is often used together with *Karma*. A baby’s death is frequently linked to a woman’s fate, which in turn is thought to map out from her *Karma*. The use of the term ‘fate’ indicates a stronger belief about the lack of control over babies’ deaths. A 31 year-old mother did not see any possibility of preventing the death of a baby against her fate:

How can we stop this [a death of a baby]? We can't prevent a man dying and a river flowing. If the baby is not in your fate (Chado), s/he will certainly die. Look, these other children, they are here. That baby was not in my fate (Chado), and passed away on the day after birth. (Interview, mothers)

A woman’s *Karma* or fate is perceived to be strongly related to her experience of perinatal deaths, particularly when she experiences continuous losses, usually the loss of two or more babies. *Karma* and fate is often perceived personally, as a woman’s personal fate. Believing that this was her personal fate, a 20 year old mother, who lost her 3 babies, even told her husband to marry another woman, and said she would not want him to be sad on account of her personal fate:

I shouldn't ruin my husband’s future. It is my fate [I lost these three babies]. I am unlucky; I have no secure future (Gharbar). I am not sure if my babies would survive if I had married another man. It is my Karma. I told him to marry another woman [to have a child]. (Interview, mothers)

The perception of *Bhagya* (fate) is even more complex. A few women perceived fate not only as a reason behind the deaths of their babies, but also as a reason for other, future consequences that they will have to bear.

Different from *Karma* and fate, the participants rationalised destiny solely to a baby’s own predestined future. A few women had no idea of the causes of their loss, and simply accepted it, nodding their head on their husbands’ comment and staying busy caring for their other babies. One man who lost 2 newborns, is an educated person, works in public office, and still he perceives that it was his babies’ *Lekhanta* (destiny) to die:

Our two babies died after birth. What to do [there is no way]. No one can control these deaths. The ones who are to die will die anyway. This was Lekhanta (the destiny) of these babies. The rest of our babies survived. Now, they are growing up. We have a grown up eight year-old daughter, a young boy and a baby girl. (Interview, family members)

The belief about destiny implies a complete lack of control over their babies’ deaths. Participants likened the babies to a cucumber (*Kakadi*), which could be picked from the kitchen garden any time before it is ripe:

I lost my own children, and also lost my three grandchildren. What disease did those little ones have? There is no other reason, just a destiny (Lekhanta). I don't know, it was neither heat (Taplagne), nor any other problems with these babies. The newborns are like cucumber (Kakadi), they could be picked up anytime as per God’s plan. One God gave it and another God took it away. (Interview, family members)

During an informal chat in the village, a local faith healer commented that *Lekhanta* (destiny) is the reason for babies’ deaths before *Chhaith*, a local Hindu ritual celebrated usually on the sixth day after birth:

When a woman is hungry, it affects her baby; we call it Hanpiyera (work exhaustion and hunger). The baby dies in the womb. If the baby dies before Chhaith (the sixth day celebration), it is due to Lekhanta (destiny). This is not due to anything related to mother, father and family. (Interview, faith healers)

The deaths of babies after birth during a woman’s confinement in the birth place, Gotha, are also attributed to destiny. The women and families were more convinced about destiny as a reason for a baby’s death when the baby died after seeking help from faith healers.

## Discussion

This study has examined the religio-cultural contexts surrounding perinatal mortality in two remote mountain villages of Nepal. These villages were selected because they ranked lowest in terms of development and child survival and provide rich evidence of local ways of understanding and responding to the very high levels of perinatal mortality, even though they might not be representative of all the villages in the region. The collective experience of perinatal death in the community has shaped the villagers’ construction of perinatal deaths as inevitable experiences which modern healthcare systems cannot address. Some people believe that modern healthcare may even make the situation worse. Individual and collective experiences are viewed as valid sources of knowledge in the social constructionist view [60]. In this study, the past experience of women and the experiences of older family members construct the knowledge of younger women. The persistent occurrence of perinatal deaths is considered as a ‘generational continuum’, bound to occur with everyone.

### Acceptance of ongoing perinatal deaths

This study has identified acceptance as the norm when a baby dies before term *(Hunemahina)*, as a stillbirth *(Hudaimareko)* or during a mother’s and baby’s confinement after birth until the third postnatal week. In this study, the women’s and families’ description of perinatal deaths as *Pakhalajane*, or *Aadanjhadne* indicates premature deaths before term are not viewed as lives lost, but perceived simply as a diarrhoeal condition as if women were emptying their bowel. Hence, these are not considered worthy to report to authorities, nor to be mourned. Such perceptions are similar to the views of Hmong women living in Australia who consider stillbirths and neonatal deaths as non-significant events [61]. A recent study from the rural Amhara and Oromiya regions of Ethiopia [62], a study from Tanzania [63] and a study from Uganda [30] have described similar perceptions of low social significance given to stillbirth and neonatal death, as they are considered as deaths of non-humans, deaths of spirits, or events not worthy of sharing with others.

A sense of stigma about perinatal deaths has been reported by some studies in African [29, 63], Asian [64, 65] and migrant Australian communities [27, 28]. These studies have discussed stigma as one of the key factors making perinatal deaths invisible in communities [9, 63, 66]. However, in the present study, such deaths did not appear to carry any stigma; they were simply considered to be of low importance, and therefore not worth reporting. The common experience of perinatal deaths among every family, who also view these occurrences as generational continua, has led them to believe that these occurrences are so common that no one pays any attention to them. Another reason for not attaching importance to such deaths is the lack of any ritual significance of a stillborn baby or neonatal death. A perinatal death is considered a mere biological loss not requiring any death rituals. Such a construction does not consider perinatal death as the loss of a social individual, which means no attention is paid to improving perinatal survival in the study villages. Loss of these early lives is more a question of a non-value attributed to their personhood in these villages, reminiscent of Aries’ argument in relation to the social construction of childhood that parents will not respond too emotionally to infants who might die early, and hence consider them as “neutral” (or non-person) for some time after birth [67]. This loss is also not considered a psychological burden to women and families to attract a stigma for having experienced such losses. Stigma-related burden is almost non-existent in these communities also because of beliefs about God and *Karma* in illness causation and death. This indicates a level of acceptance of perinatal deaths to such an extent that parents and families see no reason to take any measures to prevent such deaths.

### *Dewata* (God) as a factor in health and sickness

People believe that God exerts a powerful influence on illness causation and response to recovery. In these villages, God is omnipotent and affects every aspect of day to day life, and is not just a symbolic statue kept inside the house [23]. Such a worldview of God shares similarity with the traditional Akan religious worldview about health and sickness as described in a study from Ghana [68]. The indigenous Akan people in Ghana, and people in Tehuledere region of Ethiopia [69], believe in a host of human (witch, sorcerer), non-human (ghost, ancestor, evil spirit) and supernatural beings (God/Allah and deity) having capability to affect their lives positively and negatively. In the study villages in Nepal, common illnesses of mother and baby are also believed to be due to the displeasure of God, described under the generic name *Dewata*, and are believed to operate in different forms through human and non-human agents. The study also showed the villagers having their own illness language broadly categorised into two types: (i) illness solely attributed to God, where the cures are shaman healings, prayers, animal sacrifice, offerings, worships, mantra recitations, sprinkling holy waters, exorcism and amulets; and (ii) illness in which God is attributed as cause, yet the care combines the former approaches with the use of local herbs. In both illness types, local faith healers are the key care providers because they are believed to possess Godly powers and have the ability to invoke the mercy of God. Other studies from Nepal also describe how villagers seek the help of traditional healers for general health problems [70, 71].

Beliefs about perinatal sickness and death due to supernatural forces are also revealed in other studies in Africa and among Hmong women [31, 61, 62]. What is added from the present study is that not only baby’s illnesses but also mother’s common illnesses, birth complications and contraceptive norms are considered to be in God’s control. In the villages studied in this research, God is believed to be both a cause and a cure for mothers’ and babies’ problems along the pre-pregnancy to postnatal continuum. Birth complications which require skilled attendance or immediate referral are believed to be caused by God’s wrath, so that the family will invite faith healers to perform exorcism, prayers and offerings to God until the last minute. Importantly for healthcare provision, the study identified the belief that for a sickness due to God’s wrath, going to health facilities might negatively impact on the cure. Health workers are therefore consulted only when none of these work, and usually only after permission is obtained from the faith healers.

### Rationalising perinatal deaths as *Karma* (past deeds), *Bhagya* (fate) or *Lekhanta* (destiny)

Fatalism has been described by studies in different South Asian countries as a means to accept various events, resulting in an inertia where attitudes and behaviours perpetuate the occurrence of such events. Outsiders might view these as “risky” behaviours which must be changed to prevent the occurrence of events such as perinatal deaths. An Indian study [72] showed that belief in *Karma*, sin and God’s punishment were described as reasons for Leprosy by about two-thirds (65%) of the study’s interviewees. Fatalistic beliefs about infant deaths are identified in other studies, such as in the upper Lombok region of Indonesia where infants died due to simple treatable conditions [65]. A multi-country study from South Asia and Africa records fatalism surrounding treatment of neonatal infections [73], healthcare for small and sick newborns [74] and stillbirths [66]. However, none of the studies referred to here have thoroughly explored the specific socio-cultural contexts of the events investigated in as the way which the present study has. In these Nepalese study villages, the religio-cultural base of women’s *Karma* (past deeds) and fate were interchangeably used as reasons for a baby’s death. These beliefs intensified the acceptance and fatalism about perinatal death so that women and families remained passive about seeking healthcare for perinatal sicknesses, contributing to the continuing occurrence of these preventable deaths. One of the most common phrases during interviews and informal chats about why a baby died, was ‘What can we do? This was my Karma’ *(Ke garne*, *mero karma yestai)*, reflecting deeply rooted fatalistic beliefs. Such fatalistic beliefs came out even stronger when women rationalised their baby losses as *Lekhanta*, which they often meant as the baby’s personal destiny, thus feeling helpless in doing anything to prevent the baby’s death.

### Implications for future health provision

#### Raising critical consciousness about Karma: As a fatalistic belief to empowerment

Max Weber, in his book ‘The Religion of India: Sociology of Hinduism and Buddhism’ described the fundamental values of Hindu and Buddhist religions, *Karma* and reincarnation as doctrines of fatalism [75]. Weber states that these values do not talk about this world but about a supernatural world, and that they point to past or future lives. Although Weber’s interpretation matches the reality of the villages studied in this research, the concept of *Karma* may be viewed as a universal law of justice, a law of cause and effect. From the present study, it is argued that the perception of *Karma* and persistent occurrence of perinatal deaths in the study villages is the result of their false *Karma* consciousness. *Karma* is not meant to endure inequity or injustice as the women face in the study area of Nepal. In the *Bhagavad-Gita*, the fundamental religious textbook of Hinduism [76], *Karma* is described as a great art of performing action in all realms of thinking, speaking and acting. It is not so much about the past *Karma* of previous lives, it is mainly about present *Karma* which an individual is considered to have a control over, hence bringing the power into the individual’s authority and will. Therefore, the *Karma* doctrine is described as bringing awareness about one’s actions and bringing back cause and effect into his/her control rather than relying on fate or passively waiting for past karma to map out one’s fate. It is meant to empower one from weakness, pessimism and escapism, and to help one remain firm as a *Kshatriya*, a warrior who is mindful of the realms of his thoughts, speech and actions. The values that Weber described as fatalistic refer to teaching an art of living, and a way to liberate one’s life. On this basis, in the study villages an active collaboration could be sought with the local religious leaders and faith healers to correct the misconception (or a general popular conception) of these fundamental values and thereby potentially offering a slight reorientation of belief to local women and families. This argument might also at once reinforce their religio-cultural values yet also avoid the false perceptions and associated fatalistic views which have become popular conceptions in these areas rather than the scriptural ones.

In the present study, the acceptance and fatalism related to religio-cultural contexts is an emergent theme analysed through the participants’ narratives regarding the experiences and beliefs about their stillbirths and neonatal deaths. The deeply interconnected religious, spiritual and cultural values have been termed together as religio-cultural contexts. One may argue that the religio-cultural beliefs of *Karma*, fate and God have healed the wounds of women and families and any potential psychological burden from experiencing perinatal losses. However, the continuous human losses cannot be justified on the grounds of human rights, or the right to life of every child [77], nor when almost all of these deaths are most likely preventable. Addressing the false perceptions of *Karma* and *Dewata* (God) with due respect to local culture may be a key to the way forward.

#### Using a socio-cultural lens in reaching care: Bridging professional dialogues with lay discourses

Current perinatal survival policy and practices in Nepal describe a predominantly bio-medical discourse to improve survival, with less regard to the evidence regarding local socio-cultural contexts in the mountainous regions [12–15]. The policies are influenced by overwhelming national and international evidence on epidemiology and bio-medical risk factors of perinatal deaths: stillbirths [9, 78], and neonatal deaths [8, 79]. The available interventions are largely bio-medically oriented, aimed to prevent deaths from sickness, complications of prematurity, infection and asphyxia, based on a Western medical viewpoint of the causes. By comparison, the lay constructions and beliefs surrounding perinatal sickness and death see loss of early life as generational perpetuation and common experiences; they attribute perinatal sickness (and cure) to God; and rationalise perinatal deaths as *Karma*, fate and destiny, resulting in the belief that either no-one can intervene or only a faith healer can intervene. Both views lead to formal healthcare either not being sought at all, being sought “too late”, or being deemed as actually more harmful. The findings strongly indicate a need for bridging professional discourses with lay discourses. The healthcare systems and policies need to acknowledge and negotiate this in their actions about improving poor perinatal survival.

#### Partnership between health service providers and faith healers

The present study argues that it is not just cultural perceptions of safety with God which Kaphle [23] discussed, but it is a false awareness of God in their religio-cultural context, most likely begun as a moral order to instil discipline which became reinforced by faith healing practices. The belief in God’s influence in health and sickness has been a predisposing factor preventing families from seeing the medical severity of any sickness or childbirth complications of a mother or baby. Eventually, this influences families in who to choose as their healers [80]. They therefore choose traditional healers as the medium to invoke God, thus delaying, or indeed often preventing, them from seeking skilled and timely care from “formal healers” (in the healthcare system). Furthermore, the perception of religious beliefs about God and *Karma* are popular concepts rather than scholarly concepts. On this basis, the present study urges health professionals to work in mutual collaboration with faith healers and religious leaders so that a mother and baby could access timely healthcare for any sickness and could be saved from simple avoidable causes, and at the same time also continue to observe their religio-cultural duties. Such interventions have been introduced in Indonesia, where trained midwives work in collaboration with traditional birth attendants to attend women’s delivery and both provide for the emotional and cultural needs of local women, refer pregnant mothers, and provide post-delivery services in the communities [81].

There has been a growing realisation for the need to integrate spirituality in medicine/healthcare [82, 83]. This has been considered especially important in addressing the religiosity of patients in societies with diverse faiths. McCormick and Min [83] suggest that a spiritual history should be recorded about every patient as a part of his/her general medical history so that any religious/cultural/spiritual beliefs can be understood, and utilised as a resource for the healthcare, support and wellbeing of the patient. Although the study villages are not open societies with diverse faiths, the engendered context of the perception of God as an aetiology of sickness, and the religious value of their Karma being seen as a reason for perinatal deaths, indicates a lack of consideration of religio-cultural factors in the formal health system in the villages. It is imperative for primary healthcare workers to understand and address such beliefs in these communities in a culturally safe way, not merely focusing on instructing about “danger signs” from a medical perspective during pregnancy, birth and postnatal period to a handful of women attending health facilities. Rather, it would be imperative to revise the entire curriculum for the training of doctors, nurses, midwives and health workers to include training to address religio-cultural issues in health in specific communities.

#### Intensifying community engagement: Revisiting the contents of behaviour change communication

Ending preventable stillbirths and neonatal deaths is an international goal [84]. A range of studies [85–87] have suggested a long list of family/community and health facility based interventions to prevent these deaths. Although these interventions are typically characterised as family and community based, they are often prescriptive and do not understand the context and awareness level of women and families in the communities. Women’s and families’ construction of perinatal deaths as a natural event of low social significance means that they pay less attention to ongoing perinatal deaths. On the other hand, this indicates that they most likely have a low level of awareness and behaviour change for adopting healthy perinatal care (seeking formal health care), even though the national policy strategies [12, 13] are aimed at such behaviour change. The key bio-medical focus of the policy discourse about causes of stillbirths and neonatal deaths (infection, asphyxia and intrapartum complications) is intended to limit the content of behaviour change to conveying the knowledge of danger signs (during pregnancy, delivery and postnatal) to mothers. However, this does not link to local understandings of what constitutes danger. Local religious/faith healers could be approached to ask about the possibility of supporting women and families to explore and discuss their constructions related to personhood status of a stillborn and a newborn baby (loss of early lives as natural events of low social significance), illness causation (God) and their rationalisation *(Karma)*. Assisting communities in this way could be a key focus and content of family and community based behaviour change interventions and birth preparedness packages. Otherwise, the prescriptive list of interventions or their packages (newborn/child intervention packages) alone are likely to be much less effective, and this could be one of the reasons for its low impact in Nepal’s newborn intervention package [21, 88].

#### Addressing fatalism as a systemic issue

Målqvist [89] described the invisibility of neonatal deaths in a northern province of Vietnam due to a dysfunctional reporting system. A study from India also described that reporting the actual number of deaths is avoided by health providers as deaths could be judged as being due to their poor performance [90]. The present study suggests that the invisibility of perinatal death in this study’s remote mountain villages is reinforced by the fatalistic attitude towards perinatal deaths, not only in the community but also in the local health system. The sum total of deaths reported by participating women from the study villages provided a larger number of deaths than the sum total of the perinatal deaths reported by the District Health Office for the last four years across the 24 villages in the district [54]. This underreporting seems likely due to the low social significance accorded to perinatal deaths. Hence, the service providers and female health volunteers did not find it worth inquiring and reporting perinatal deaths. This systemic fatalism (including among community health workers) has contributed to the invisibility which is certain to reduce the efforts to implement perinatal survival programmes by the local health system. Such rural areas as in Nepal are therefore likely to be overlooked.

#### Methodological implications

The study has also a methodological advantage. A large body of evidence in perinatal survival research is based on structured surveys and verbal autopsy [91] to describe a pattern of mortality and service utilisation across socio-demographic determinants and medical causes of deaths. By comparison, this study has examined the influence and interactions in socio-cultural contexts that lead to perinatal deaths. Recently an interest is growing about the need for social autopsy, which analyses death narratives and focuses on identifying social aspects impacting perinatal deaths [92]. This research has found that in-depth qualitative interviews with women and their families who have experienced perinatal deaths are possible and are an appropriate way to uncover the influences of the local socio-cultural context that contribute to poor perinatal survival. In addition, women and families in the villages indicated that being interviewed made them feel respected and valued their experiences. The limitation of this study is that the interview participants were selected purposively; the views of others may be different, such that the study lacks a wider generalisability in other regions. However, the study has provided a thorough examination of the religio-cultural context of perinatal death in the study region and has identified policy implications in implementing interventions to address the ongoing high stillbirth and neonatal death rates in rural villages.

## Conclusion

Perinatal deaths are regarded as common occurrences, and religio-culturally constructed as the deaths of foetuses and neonates which have no personhood status or value. Mothers’ and babies’ sickness and recovery is attributed to *Dewata* (God)’s will, while Karma (past deeds), *Bhagya* (fate) or *Lekhanta* (destiny) are perceived as reasons behind perinatal deaths, which are religio-cultural beliefs deeply rooted in the mountain communities. These fatalistic beliefs are perceived as culturally safe, yet pose a high risk to the survival of babies when the bio-medical evidence suggests that 99 percent of perinatal deaths are preventable. The persistence of fatalism also raises the question of whether perinatal survival interventions have effectively reached the rural communities and villages. The findings of this study strongly indicate the need to invite discussions on the construction of personhood and social significance of foetus and newborn babies in the current behaviour change discourse. The findings further call for starting partnerships of medical health practitioners at primary health care level with faith-based healers and religious figures so that ways can be found to respect the lay worldviews of *Dewata and Karma* in health and sickness without these continuing to perpetuate poor perinatal survival in the mountain villages.

## Supporting information

S1 File Brief description of study participants. (DOCX)

S2 File Interview guides for women, service providers and stakeholders. (DOCX)
