# Supplementary material for: Religio-cultural factors contributing to perinatal mortality and morbidity in mountain villages of Nepal: Implications for future healthcare provision
Source: PLoS One. 2018 Mar 15;13(3):e0194328. doi: 10.1371/journal.pone.0194328 (PMC5854484; doi:10.1371/journal.pone.0194328)
Supplement: S1 File — (DOCX) [file pone.0194328.s001.docx]

**Study Participants**

| **Participants** | **Number of Interviews** | **Details** |
| --- | --- | --- |
| Women and family members | 42 | The family members included husbands, mothers-in-law, fathers-in-law, father, and sisters-in-law. Six interviews were mainly of husband’s views with added comments from women. One was with only mother-in-law. In other interviews, as well as the woman, husbands, fathers-in-law, mothers-in-law as available in the interview setting added their comments during the conversations. All families had gone through perinatal losses (stillbirths and/or neonatal deaths). A majority were recent deaths, in the last four years. |
| Health service providers | 11 | Nine were Skilled Birth Attendants (SBAs), one Auxiliary Health Worker, and one non-SBA trained Auxiliary Nurse Midwife. |
| Female Community Health Volunteers (FCHVs) | 2 | One FCHV had lost her newborn grandchild in the last two years, and one had given birth the last year. |
| Stakeholders | 4 | One teacher, two local journalists, one Non-Governmental Organization officer |
| Support staff at health institutions | 2 | One support staff from a community birthing centre, and one from the local health institution in the village |
| Traditional Healer | 1 | The locally popular head of traditional healers who treats the largest number of children across the villages |
