# Supplementary material for: Religio-cultural factors contributing to perinatal mortality and morbidity in mountain villages of Nepal: Implications for future healthcare provision
Source: PLoS One. 2018 Mar 15;13(3):e0194328. doi: 10.1371/journal.pone.0194328 (PMC5854484; doi:10.1371/journal.pone.0194328)
Supplement: S2 File — (DOCX) [file pone.0194328.s002.docx]

**IN-DEPTH INTERVIEW GUIDE**

(Expected time duration 30 to 40 minutes)

The Socio-cultural and Healthcare Context of Perinatal Survival in Rural Mountain Villages of Nepal

___________________________________________________________________

1. **Women’s Interview**

***Socio-demographic profile***

1. Just to start our discussion, perhaps you can tell me about yourself- you and your family?

(Probe: completed age (years), marital age, current marital status, family size, caste/ethnicity, participant’s education, husband’s education, personal habits (drinking, smoking) and so on.

1. Can you tell me how you and your family manage to live here?

(Probe: living and working conditions, occupation of participant, her husband and other key family members, household wealth (major income source, land availability, food sufficiency, means of transport, housing type, toilet facility, kitchen-indoor air etc.] ; Interviewer Notes: needs general observation also…)

***Maternal characteristics***

1. Can you briefly describe to me your childbearing history?

(Probe: age at first birth, gravida/parity including losses, current pregnancy, previous spacing, intended/unintended births including any incidents of family violence, family planning,)

***Perceptions about care seeking and survival***

1. What do you think (believe) about seeking healthcare during pregnancy, during labour/birth, immediately after the birth for mother/baby, during the early weeks? Are there any special situations? Why? Why not?)
2. Have you heard of any things that the mother, family or health worker or anyone else can do to make it more likely that the baby is born healthy and survives?

(Probe : beliefs, knowledge, health system, acceptance of the formal care: Antenatal visits, TT immunization, Iron intake, Birth preparedness (have money, transportation, known to Skilled Birth Attendant and health facility, postnatal care – clean cord cutting, breastfeeding, immediate drying/warmth etc.)

1. Can you tell me about things that you or others do to care for the baby as soon as it is born, within the first month?

(Probe: health behaviours, acceptance of the formal care: Immediate neonatal care practices (drying/wrapping, keeping warm/cool, breastfeeding, cord-care, delay bathing); new-born care visits, pre-lacteal feedings)

When do you think the baby is sick or baby is in difficult condition? What do you think can be done when the baby is sick? Where to take it for help? Why? Why not?

(Probe: common neonatal danger signs (infection-diarrhoea, pneumonia, unable to suckling breast milk, fever...see protocol), any time or situation that these differ e.g. times of day/year (greater need to keep warm in the cold winter months?); times when harder/easier e.g. to breastfeed…)

***Experience of care***

1. Can you describe to me your experience of the care you received during your last pregnancy, the last birth and after the birth up until the first month?

(Probe: care at home, care at health facility, birth preparedness-knows and decides where to deliver, money, transportation, and support from family] Note: focus also on other than the last pregnancy if it is interesting and relevant to the phenomenon-i.e. loss, sickness &/or small sized baby

***Experience of sickness/stillbirth/newborn death***

1. Can you share me about your most recent story of losing a baby (or having a new baby who is small or sick)?

What was it? When did it happen?

Did you go anywhere to prevent/treat it? Do you have any ideas about why it happened?

Do others think or say anything about why they think it happened? (For example by husband? mother-in-law? health workers?)

(Probe: the event (perinatal death, neonatal death, sick/small sized new-born), Interviewer Note: this is the central aim, take time in in-depth probing of the story)

1. (Only for mothers whose babies are/were sick). Would you describe how you cared (or caring now) for your baby while it is/was sick?

What was the care specifically-what, where & when? Care at home? Contacted other traditional healers? Facility based care? Contacted female health volunteers, health facility and hospital? Private nursing home?)

If no facility-based care, why?

(Probe: Response to the event)

1. Do you think this particular event (referred to either 8 &/or9) could have been prevented in anyway? How? *(ask with an intention to help other women, assure not to make the woman feeling guilty about her lose)*

(Probe: role of husbands, mother in laws, other family members, female health volunteers and health workers, Expectations from family (husband, mother in law) and facility-based institutional care)

1. In relation to the event (8 &/ or 9) did you have any chance to consult a health facility or to have home visits from health workers/volunteers/health workers? How did you experience it?

(Probe: the care from female health volunteers, health workers-doctor, nurse, referral and compliance-if not why? Care from traditional healers?), the quality of care, compliance, referral, constraints/enablers in access to care)

Do you think the care you received (or didn’t receive) contributed in any way to this event happening or to preventing it being worse? How?

1. Can you tell me about any other problems that you experienced during pregnancy? Childbirth? When you were pregnant with the baby (related to the event above—Sickness, Stillbirth or Neonatal Death)

(Probe: health problems, experience of delays-seeking/receiving care, Note: focus also on other pregnancies and if similar previous events had occurred in the woman’s life provided it is interesting and relevant to the phenomenon-i.e. loss, sickness &/or small sized baby)

1. Did you find any other women having the similar problem (Sickness/Stillbirth/Neonatal Death) in your community?

(Probe: Other similar events in the community and opinion on prevention)

Why do you think that they had to experience such events?

Do you have any ideas about what might help prevent this situation happening again for yourself or your neighbours?

1. Do you have any other comments you would like to add, or any questions you would like to ask?
2. **Family Members (husbands, mothers-in-law, fathers-in-law)**

(Additional questions to Section A, as relevant)

1. Can you tell me about any other problems that you as a family member experienced about her (the woman’s) pregnancy? Childbirth? When she was (the woman) pregnant with the baby (sick, stillbirth, newborn death)
2. Do you have any different observations/views about the loss of baby? Healthcare seeking? Anything else?
3. **Female Health Volunteers**
4. What do you think the ways you can contribute as a volunteer to make it more likely that the baby is born healthy and survives?

(Probe: her perceived roles)

1. Can you share with me the family’s [referred to the particular woman] most recent story of losing a baby (or having a new baby who is small or sick)?

What was it? When did it happen?

Did they go anywhere to prevent/treat it? Do you have any ideas about why that happened? Could you help this family? How?

(Probe: the event (perinatal death, neonatal death, sick/small sized new-born), Interviewer Note: this is the central aim, take time in in-depth probing of the story)

1. (Only if the family had sick & or small sized baby). Would you describe how you supported to care (or caring now) for the baby while it is/was sick/small sized?

What was the care specifically-what, where & when? Care at home? Contacted other traditional healers? Facility based care? )

If no facility-based institutional care, why?

(Probe: Response to the event)

1. Do you think this particular event (referred to either 2 &/or 3) could have been prevented in anyway? How? *(ensure that there is no blame to each-others, rather let her fully explain the story)*

What did the family could do to prevent it? Was that enough? Why not? Why do you think that they could not do it?

(Probe: your role and family’s expectation of facility-based institutional care, barriers and enablers, health volunteer’s perception of traditional and institutional care)

1. Can you tell me about any other problems that you as a female health volunteer experienced about her (the woman’s) pregnancy? Childbirth? When she was (the woman) pregnant with the baby (related to the event above)

(Probe: experience of delays-seeking/receiving care, Note: focus also on other pregnancies and if similar previous events had occurred in the woman’s life provided it is interesting and relevant to the phenomenon-i.e. loss, sickness &/or small sized baby)

1. Did you find any other women having the similar problem (the events) in your community?

(Probe: Other similar events in the community and opinion on prevention)

Why do you think that they had to experience such events?

Do you have any ideas about what might help prevent this situation happening again for yourself or your neighbours?

1. Do you have any other comments you would like to add, or any questions you would like to ask?
2. **Health Service Providers and Other Key Informants**
3. Can you describe your experience of working as a health worker with pregnant women and new mothers?

[Probe: years of experience, specific trainings received in maternity and newborn care, work duration in particular village/health facility]

1. Can you describe what sorts of services this health facility provides?

[Probe: provide to moms and new-born? range of care and services delivered from the facility]

1. In what different ways are you contributing in providing essential care to new-borns from this health facility?

[Probe: perceived roles, skills, knowledge: immediate care of babies after birth, care for low birth weight babies, care during sickness: managing infections-diarrhoea, pneumonia, resuscitation……..]

1. Data shows the situation of survival of newborn babies in the mountain region is worst in the country. Mugu is also considered as a mountain district with poor child/newborn survival, what do you think the reasons behind?

(Probe: care seeking for women-babies, quality of care from health facilities)

1. Do you think any aspects of the healthcare system contribute to these events despite people’s efforts?

[Probe: healthcare skills, training, resources supports available to health facility, referral and related problems, incentives/motivation to work, supports from supervisory institution; Why? Why not?]

1. Do you think these events could be prevented in anyway?
2. Are women in this district/community seeking care during their pregnancy/delivery/postnatal period for themselves and their babies? How is this trend from health facility? If not so encouraging, why? (Probe: the perspectives of health worker on enablers, constraints, care at home, traditional care, facility-based institutional care)
3. Can you share with me the last event [referred to the particular one from the woman’s interview or any other event] ---most recent story of losing a baby (or having a new baby who is small or sick)?

What was it? When did it happen?

Did they go anywhere to prevent/treat it? Do you have any ideas about why that happened? How did you help this family?

(Probe: the event (perinatal death, neonatal death, sick/small sized new-born), Interviewer Note: this is the central aim, take time in in-depth probing of the story)

1. Do you think this particular event could have been prevented in anyway? How?

What did the family could do to prevent it? Was that enough? Why not?

What was the role of your health facility?

[Probe: your expectations from the family? observed any delays/constraints at home, community level, facilitation of the case at health facility] The policy/strategy vs local health context; socio-cultural context

Do you have any ideas about what might help prevent this situation happening again for the woman/family or anyone in this community?

1. Do you have any other comments you would like to add, or any questions you would like to ask?

**THANK YOU SO MUCH FOR YOUR TIME AND SUPPORT.** ###
